# Single-cell Co-expression Subnetwork Analysis

**DOI:** 10.1038/s41598-017-15525-z

**Published:** 2017-11-08

**Authors:** Thomas E. Bartlett, Sören Müller, Aaron Diaz

**Affiliations:** 1 Department of Statistical Science, University College, London, UK; 2 Department of Neurological Surgery, University of California, San Francisco, USA

## Abstract

Single-cell transcriptomic data have rapidly become very popular in genomic science. Genomic science also has a long history of using network models to understand the way in which genes work together to carry out specific biological functions. However, working with single-cell data presents major challenges, such as zero inflation and technical noise. These challenges require methods to be specifically adapted to the context of single-cell data. Recently, much effort has been made to develop the theory behind statistical network models. This has lead to many new models being proposed, and has provided a thorough understanding of the properties of existing models. However, a large amount of this work assumes binary-valued relationships between network nodes, whereas genomic network analysis is traditionally based on continuous-valued correlations between genes. In this paper, we assess several established methods for genomic network analysis, we compare ways that these methods can be adapted to the single-cell context, and we use mixture-models to infer binary-valued relationships based on gene-gene correlations. Based on these binary relationships, we find that excellent results can be achieved by using subnetwork analysis methodology popular amongst network statisticians. This methodology thereby allows detection of functional subnetwork modules within these single-cell genomic networks.

## Introduction

Systems which can be modelled as networks are ubiquitous. Well-known examples include social and economic networks, as well as many examples in cell biology such as gene regulatory and protein signalling networks. Much work has been done in recent years to better understand the theoretical properties of network models. This has lead to rapid advances in the power, applicability and computational efficiency of these methods. In the biological setting, gene co-expression networks^1^ have proved very popular. Many methods exist to carry out gene co-expression network inference and analysis; these methods are typically based on microarray data. For a review of popular gene co-expression network methods, with their strengths and weaknesses, see^2^.

In recent years cell biology has experienced a rapid growth in use of single-cell gene-expression data. However, not much work has been done so far to design and adapt network models specifically for such data. Challenges to developing network models in this context arise because single-cell gene-expression data are typically very noisy, and furthermore suffer from zero-inflation^3^. Both these effects result in large part from sequencing read-depths being kept relatively low, in order to keep experimental costs down. At low sequencing read-depth, heterogeneity between cells (i.e., biological variation) as well as random effects in the sequencing processes (i.e., technical variation) mean that low-expressed genes may either be absent from the data (zero-inflation) or subject to much noise. A way of overcoming these problems is to do a model-based adjustment of the statistics (such as correlations) which are calculated from the gene expression counts^3^. Another possibility is to model only those genes which are robustly expressed across cells. There is much potential for useful work to be done to combine powerful network models with single-cell gene expression analysis.

A typical problem in network modelling is how to identify subnetworks or groups of network nodes within the network. This problem is very similar to the problem of clustering network nodes, or in the context of gene expression network modelling, clustering genes. Clusters of genes, or gene subnetworks, are modules of genes which are tightly co-regulated, or which regulate each other, or which carry out specific biological functions. One of the most popular methods of clustering in gene expression analysis is agglomerative hierarchical clustering. This method typically considers similarity between genes in terms of their correlation across samples (‘correlation distance’). However, hierarchical clustering often performs badly in challenging contexts, when it will frequently assign most of the genes to one big cluster (as we will show in the next section). This problem is made worse when the correlations between genes are reduced by noise, a standard problem in single-cell data. Several alternative clustering and subnetwork detection methods also exist, which are also based on gene-gene correlations, but which are much more powerful. These include PAM (partition around medioids)^4^, and spectral clustering^5^ (i.e., *k*-means clustering in a reduced eigenspace of the expression-correlation matrix). Detailed studies need to be carried out to adapt and compare popular and powerful methods for clustering and subnetwork detection for the context of single-cell data.

Some of the most popular methods of subnetwork detection are in the class of ‘community detection’ methods. Much work has been done in recent years to understand the theoretical properties of these methods. In particular, community detection methods such as the degree-corrected stochastic blockmodel (DCSBM)^6–9^ have been found to be very effective. The DCSBM can also be viewed as a refinement of spectral clustering^5^. However, rather than starting with a correlation matrix, the theory behind such methods generally requires the strength of association between network nodes to be specified in terms of a binary-valued adjacency matrix. It is possible to obtain such a binary-valued adjacency matrix directly from the correlation matrix by mixture-modelling^10^, specifying a zero-mean mixture model component and a non-zero-mean component. Using mixture-modelling in this way also helps to relieve some of the problems which result from the correlations being reduced by noise in single-cell gene-expression data. This is because, in such a mixture model, these decreased correlations still get assigned to the non-zero-mean mixture component. Hence, these reduced correlations still lead to a ‘1’ in the corresponding binary-valued adjacency-matrix (which then represents the relationships between genes). Binary-valued adjacency matrices also have the advantage of simplifying the network, thus aiding interpretation and identifiability. The inferred adjacency matrix tends to be very sparse (typically fewer than 5% of the possible edges are present when inferred in this way, and often far less). This prioritises a small number of very important gene-gene interactions for consideration in downstream analyses. For single-cell data, community detection methods such as the DCSBM are powerful alternatives to more popular clustering methods, and the binary-valued adjacency matrix has a number of advantages for representing relationships between genes.

When a binary-valued adjacency matrix is inferred from expression data alone, an edge may represent a direct physical interaction between the product of one gene and the DNA of another, or it may represent an indirect interaction. Such indirect interactions may be via an intermediate gene, which is regulated by the first gene and regulates the second gene: this is sometimes called a ‘transitive edge’ in the network. An indirect interaction could alternatively be mediated by an epigenomic process or non-coding RNA, whereby the first gene is again involved in regulating this process, and the process is in turn involved in regulating the second gene: this again corresponds to a transitive edge. Other possibilities for indirect interactions include both genes being co-regulated by a third gene, or transcriptional influence via a *cis*-regulatory element such as an enhancer site.

In this paper we compare several different clustering and subnetwork detection methods, based on gene-gene correlations in single-cell gene-expression data. We also compare several methods of obtaining these correlations, and we assess the effect of restricting the resulting network to only those genes which are robustly expressed (i.e., are non-zero in a sufficient proportion of cells). By inferring a binary-valued adjacency matrix from gene-gene correlations, then fitting the powerful DCSBM to infer subnetworks or clusters, we are able to achieve excellent results. Using these methods, we then investigate the biological relevance of some of the most significant subnetworks which we detect.

## Results

In this section, we compare four methods to infer subnetwork modules/gene clusters from gene co-expression networks:

1. Agglomerative hierarchical clustering based on the correlation matrix (‘Hclust-cor’)
2. PAM clustering based on the correlation matrix (‘PAM-cor’)
3. *K*-means clustering in a reduced eigenspace of the expression correlation matrix (‘PCA-cor’)
4. DCSBM community-detection based on the inferred adjacency matrix (‘DCSBM-adj’)

We carry out this subnetwork inference separately for two different cell-types: neurons, and outer-radial glia (oRG, a type of neural stem cell). We do this using a publicly-available neuro-developmental single cell transcriptomic data-set^11^. The subnetwork inference is based on Pearson correlation matrices, by default. We compare the results based on Pearson correlations with equivalents based on Spearman correlations (which do not assume Gaussianity or linear relationships between the gene-expression levels). We also compare the results with equivalents based on Pearson-correlations adjusted for the effects of zero-inflation by the SCDE method^3^. In addition, we assess the effect on the methods of restricting the networks/correlation matrices to only those genes which are robustly expressed across cells. To do this, we filter genes for inclusion in the correlation matrix, and include only those which are expressed (i.e., are non-zero) in at least a specific threshold percentage of cells. We set these thresholds as 5%, 25%, 50% and 75% of cells. This thresholding leads to networks of size 8027, 3314, 1190 and 387 nodes (i.e., genes) respectively for the neuron data, and 9172, 5202, 2110, and 716 nodes respectively for the oRG data. Based on these networks of four different sizes, each of which are estimated with different ways of calculating the correlations, we compare the four subnetwork detection/clustering methods described. To make a fair comparison between the different clustering/subnetwork detection methods, we seek the same number of clusters/subnetworks for a network of given size. Full details of how this number of clusters is selected is given in the methods section.

The comparison of the methods is assessed by a metric which quantifies how biologically significant are the detected subnetworks, in terms of the extent to which they overlap with modules of co-regulated genes. We calculate this metric by carrying out gene-set enrichment analysis (GSEA)^12^, using the ‘transcription factor targets’ (TFT) and ‘micro-RNA targets’ (MIR) gene-sets available from the Broad Institute’s Molecular Signatures Database (MSigDB). Each of the 615 TFT gene-sets consists of a list of genes which share a transcription factor binding-site, and each of the 221 MIR gene-sets consists of a list of genes which share a 3’-UTR micro-RNA binding motif. The way we do the assessment to compare the methods, is by testing the overlap between each detected subnetwork with each of these 615 + 221 regulatory gene-sets. Each comparison between detected subnetwork and gene-set is carried out with a Fisher’s exact test (hypergeometric test), leading to a hypothesis-test *p*-value. These significances can then be compared across the different methods, for networks of the same size.

### Comparing subnetwork detection methods

To compare the subnetwork detection methods, we assess how biologically significant are the detected subnetworks, in terms of the extent to which they overlap with modules of co-regulated genes. For this comparison, for a given choice of method, we sum over all subnetworks and clusters the total number of significantly overlapping gene-sets (according to Fisher’s exact test with FDR *p* < 0.05), and we also report the significance of the most significant overlap for any subnetwork/cluster. The sum statistic quantifies the extent to which co-regulated genes are grouped together in subnetworks, and the most-significant statistic quantifies how precisely, in the best case, the detected subnetwork reproduces a co-regulated module. Hence, we use the sum-statistic and most significant statistic of significantly overlapping regulatory gene-sets, as complementary metrics for the comparison of methods.

In some cases, one very large subnetwork is detected (often containing at least half the nodes of the network). This is undesirable, and therefore we judge this effect to be a failing of the method. However, such large subnetworks often overlap very significantly with very many gene-sets, because the significance of Fisher’s exact test increases as the size of the overlapping groups increases. For this reason, we also report a ‘size factor’ statistic, which quantifies the greatest observed size of the detected subnetworks in relation to their expected size (under an even division of the network):

1 $${\rm{size}}\,{\rm{factor}}=\mathop{{\rm{\max }}}\limits_{k\in \mathrm{\{1,...,}K\}}\,[\frac{|{S}_{k}^{(o)}|}{|{S}_{k}^{(e)}|}],$$

where $|{S}_{k}^{(o)}|$ and $|{S}_{k}^{(e)}|=N/K$ are the observed and expected sizes of subnetwork *S*_*k*_, *N* is the total number of nodes, and *K* is the total number of subnetworks. We use the ‘size factor’ statistic as a metric to determine when the regulatory gene-set overlap statistics have been inflated by giant clusters.

**Figure 1:**
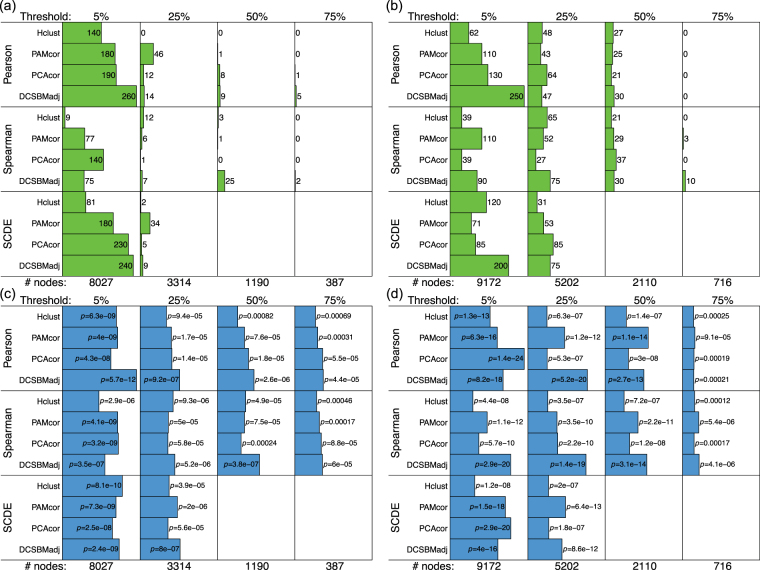
Performance of the methods: transcription factor targets. (**a** and **b**) show the total number of significant overlaps (FDR $p < 0.05$) and (**c** and **d**) show the most significant overlap ($-10\,\mathrm{log}(p)$), between detected subnetworks and the ‘transcription factor target’ regulatory gene-sets, for neuron and oRG respectively. The robustness thresholds are shown above each panel (i.e., the minimum percentage of cells in which a gene must be non-zero for it to be included in the network), with corresponding numbers of network nodes below.

**Figure 2:**
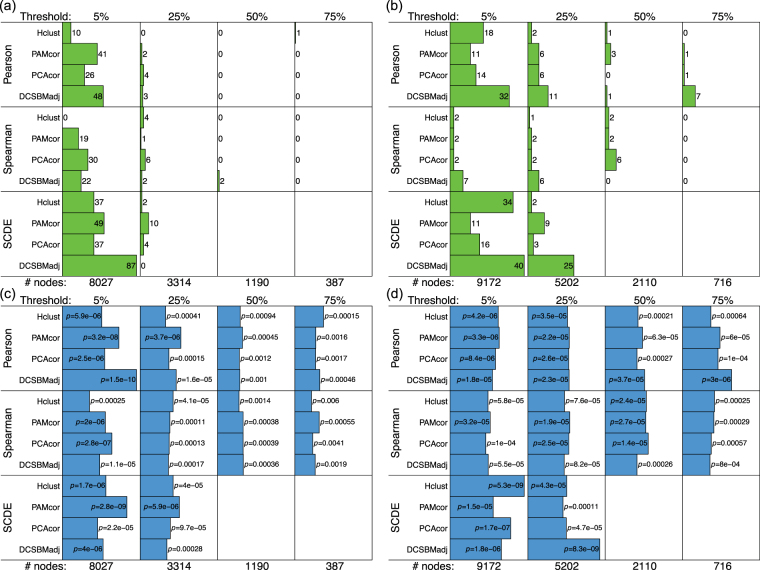
Performance of the methods: micro-RNA targets. (**a** and **b**) show the total number of significant overlaps (FDR $p < 0.05$) and (**c** and **d**) show the most significant overlap ($-10\,\mathrm{log}(p)$), between detected subnetworks and the ‘micro RNA target’ gene-sets, for neuron and oRG respectively. The robustness thresholds are shown above each panel (i.e., the minimum percentage of cells in which a gene must be non-zero for it to be included in the network), with corresponding numbers of network nodes below.

**Figure 3:**
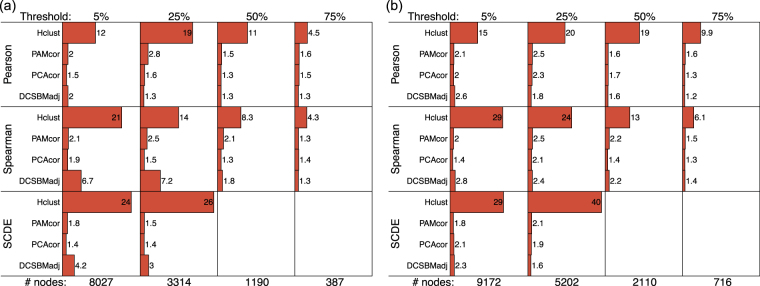
Performance of the methods: size-factors. The size factors (Equation 1) are shown for (**a**) neuron and (**b**) oRG for the different methods and network sizes. The robustness thresholds are shown above each panel (i.e., the minimum percentage of cells in which a gene must be non-zero for it to be included in the network), with corresponding numbers of network nodes below.

Figures 1 and 2 show the overlap statistics for the transcription factor target regulatory gene-sets, and the micro-RNA target gene sets, and Fig. 3 shows the size factors, for each choice of method for the neuron and oRG data. SCDE results are omitted for the network sizes defined by 50% and 75% robustness thresholds, as the SCDE method is not applicable to networks smaller than 2000 nodes.

There is a clear and definitive pattern of the DCSBM-adj method performing better, in these results. This pattern is consistent across networks of different sizes, for both the TFT and MIR assessments. Specifically, in terms of the the sum-statistics, the DCSBM-adj method performs better in 10/16 comparisons, and in terms of the most-significant statistics, the DCSBM-adj method performs better in 11/16 comparisons. When the DCSBM-adj method does not perform better, it typically performs almost as well as the best method. Also for the DCSBM-adj method, by using Spearman correlation or SCDE-adjusted Pearson correlation there is typically no improvement over using the standard Pearson correlation. Specifically, in terms of both the sum-statistics and the most-significant statistics, for the DCSBM-adj method the results are better in 8/16 cases with Pearson correlation. Similarly for the other methods, there is no clear pattern of improvement over standard Pearson correlation when using either Spearman correlation or SCDE-adjusted Pearson correlation. We note that for Hclust-cor, the size factor is typically in excess of 10, suggesting giant clusters are being detected. Therefore, the H-clust-cor method is not included in the above assessments.

For the DCSBM-adj method, we chose to infer the adjacency matrices by fitting a mixture-model. A popular alternative^13^ for inferring a network from a correlation matrix is simply to threshold the correlation matrix, e.g. at correlation-coefficient $\rho \,\mathrm{=}\,\mathrm{1/2}$ or $\rho =\mathrm{1/3}$. However, we found that the mixture-model approach is more effective in the context of the analyses presented here. Figure S1 shows analyses equivalent to those shown in Figs 1 and 2, comparing subnetwork inference using the DCSBM following network inference by mixture-modelling (DCSBM-adj) and by correlation thresholding (DCSBM-thresh). We also note that to threshold correlations in this way is somewhat arbitrary, and leaves room for for user choice and therefore also user bias. Our proposed mixture-model avoids those problems, as well as showing better performance here.

### Biological relevance of detected subnetworks

We confirmed the biological relevance of detected subnetworks with an independent neural single-cell transcriptomic data-set^14^. In this data-set, genome-wide transcriptome measurements are available for cells of various types, which are known. For this analysis, we use the data for 281 cells in this data-set comprising neurons, astrocytes, oligodendrocytes, OPCs (oligodentrocyte precursor cells) microglia, and endothelial cells. The original presentation of these data^14^ also provides disjoint (i.e., non-overlapping) gene-sets which are co-expressed in, and are characteristic of, these cell types. Therefore, we would expect that co-expression subnetworks identified for these cells would largely correspond to these cell-type specific gene-sets. This provides a method of validating the biological relevance of the subnetworks detected by the methods presented here.

**Figure 4:**
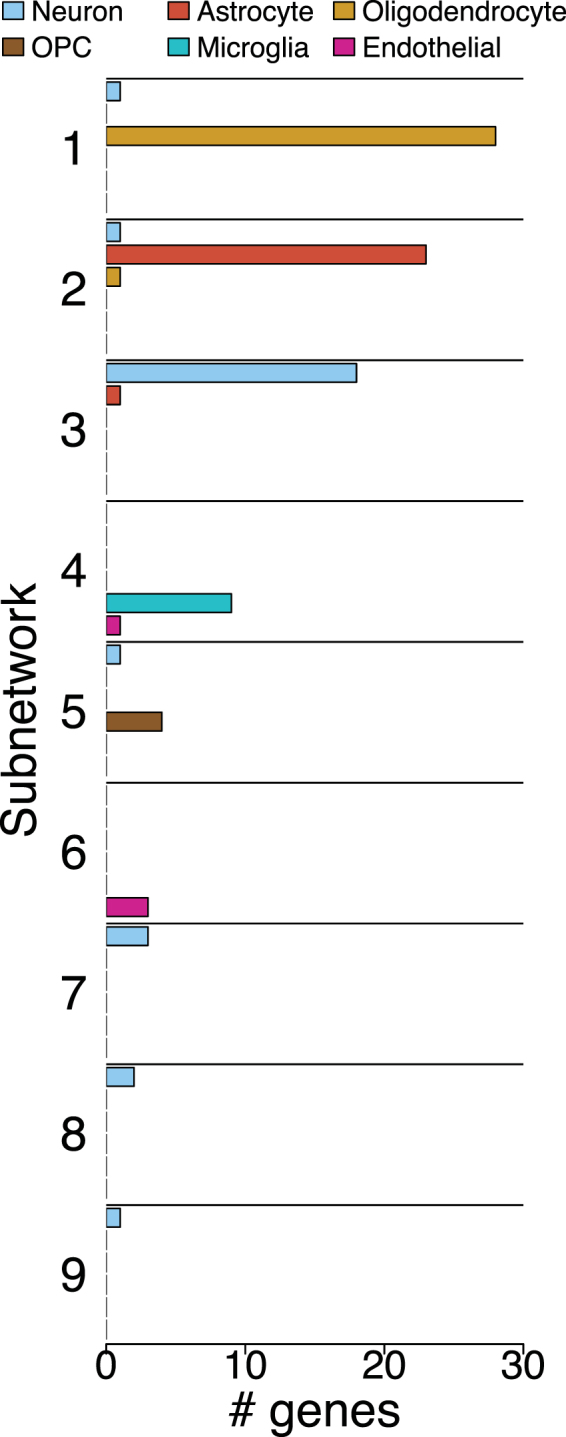
Distribution of cell-type specific gene-sets between co-expression subnetworks. For the DCSBM-adj method.

The co-expression network for this data-set was divided into *k* = 9 subnetworks (verified by the scree-plot method). Figure 4 shows how the 27 neuron, 24 astrocyte, 29 oligodendrocyte, 4 OPC, 9 microglia and 4 endothelial genes are distributed between these subnetworks, for the DCSBM-adj method. Equivalent plots for the PAM-cor and PCA-cor methods are shown in Figure S2. The Hclust-cor method failed again as it simply identified a giant cluster. The ‘normalised mutual information’ (NMI) is a statistic which can be used to quantify the accuracy of the assignment of genes to subnetworks here, compared with the original assignment of these genes into cell-type specific gene-sets. The NMI quantifies the ‘correlation’ between different assignments of the same genes to different groups or clusterings. $NMI=1$ means that there is perfect correspondence between these different assignments, and $NMI=0$ means that there is no correspondence. Here, for the DCSBM-adj method we have $NMI=0.80$, for the PAM-cor method we have $NMI=0.73$, and for the PCA-cor method we have $NMI=0.73$. Hence, the DCSBM-adj method is effective at blindly identifying subnetworks which correspond to known gene-sets in these data. Further, the DSCBM-adj method is confirmed as being more effective than the PAM-cor, PCA-cor and Hclust-cor methods. These results demonstrate the biological relevance of the subnetworks detected, particularly by the DCSBM-adj method.

### Biological importance of detected subnetworks

**Figure 5:**
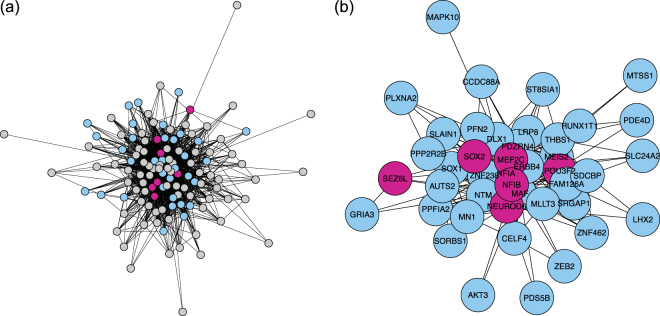
Neuron subnetwork detected in the neuro-developmental data. (**a**) Full subnetwork. Genes in the gene-set defined by the promoter sequence AACTTT are coloured blue; genes which are also important for neuronal identity are coloured purple. Other genes are coloured grey. (**b**) The component of the subnetwork which overlaps with the AACTTT gene-set.

**Figure 6:**
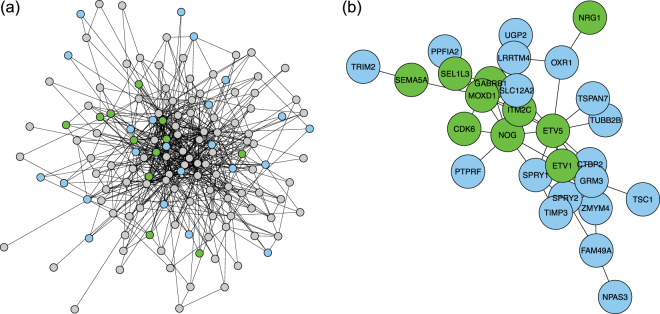
Outer radial glia subnetwork detected in the neuro-developmental data. (**a**) Full subnetwork. Genes in the gene-set defined by the promoter sequence AACTTT are coloured blue; genes which are also important for oRG identity are coloured green. Other genes are coloured grey. (**b**) The component of the subnetwork which overlaps with the AACTTT gene-set.

The most significant detected subnetworks were tested further for biological importance. The DCSBM-adj method was again chosen for this analysis due to its good performance, and because of the simplicity and identifiability of the subnetworks it infers. The number of subnetworks/clusters, *k*, was chosen as *k* = 61 by the scree-plot method. Figure 5 shows a detected subnetwork which overlaps highly significantly (FDR-adjusted $p=3.4\times {10}^{-8}$) with the gene-set defined by the promoter sequence AACTTT (marked in blue in the figure) in the neuron data. This subnetwork also overlaps with several genes important for neuronal identity (marked in purple in the figure). Figure 6 then shows a detected subnetwork which also overlaps significantly (FDR-adjusted $p=1.89\times {10}^{-3}$) with the gene-set defined by the promoter sequence AACTTT (again marked in blue the figure) in the oRG data. This subnetwork also overlaps with several genes important for outer radial glial (oRG) identity (marked in green in the figure). We note that the overlap of the AACTTT gene-set with the oRG subnetwork has a less extreme level of significance than with the neuron subnetwork (where it has the most significant overlap). From these findings, we can infer that the AACTTT promoter sequence may define a regulatory interaction which is very important for the specification and/or differentiation from oRG to neuron. Interestingly, the AACTTT promoter sequence does not match any known transcription factor, although it has previously been found to be important in foetal development^15^. Therefore, the promoter sequence AACTTT may define a regulatory interaction which is important to neuronal identity and which has not yet been described.

To cross-check the importance of this promoter sequence AACTTT, a differential expression analysis was performed using LIMMA/edgeR^16,17^. This analysis was carried out for each of 8 cell types: projection neuron, interneuron, intermediate progenitor, microglia, neural stem cell, oligodendrocyte precursor cell, outer radial glia, and pericyte. For each cell type, the top 500 most significantly differentially expressed genes were identified, by comparing that cell-type with all the other cell-types. Then, for each cell-type, these 500 cell-type specific genes were tested for overlap with each of the 615 TFT regulatory gene-sets again using Fisher’s exact test (i.e., gene-set enrichment analysis). For three of the cell-types, namely, projection neuron, interneuron and outer radial glia, the gene-set defined by the promoter sequence AACTTT was the most significantly enriched gene-set out of all 615 tested (FDR-adjusted $p=7.6\times {10}^{-21}$, $p=1.11\times {10}^{-18}$ and $p=3.07\times {10}^{-27}$ respectively). Then, comparing only the neurons with only the oRG cells, the top 500 most differentially expressed genes were similarly identified, and again testing these 500 genes for overlap with the 615 TFT regulatory gene-sets, the AACTTT gene-set was again found to be the most significantly enriched (FDR-adjusted $p=7.67\times {10}^{-25}$). Hence, the promoter sequence AACTTT is independently confirmed by this analysis to be potentially very important in the regulation of outer radial glial and neuronal identity, and possibly also in the transition between these cell-types. Finally, it has been shown previously that certain genes are important for determining the identities of both oRG and neurons^11^, including NPY, RTN1, CTNND2, SEZ6L, and NRCAM. Interestingly, two out of five of these genes, CTNND2 and SEZ6L, appear in the gene-set defined by the promoter sequence AACTTT ($p=0.030$, Fisher’s exact test).

## Discussion

In this study we have found that in the single cell genomics context, the DCSBM (degree-corrected stochastic blockmodel) performs very well. Specifically, it provides biologically relevant results, that reflect common regulatory mechanisms shared between genes. However, powerful community detection methods such as the DCSBM are still relatively rarely used for clustering/subnetwork detection in cell biology, despite the very large amount of work done by the mathematical statistics community to understand their theoretical properties. There are theoretical reasons why methods such as the DCSBM are not used more often in cell biology; for example, these methods often assume a binary-valued network representation rather than a continuous-valued correlation matrix or equivalent. We overcome this particular problem here with an empirical-Bayes mixture model, to estimate the binary-valued adjacency matrix from the correlation (or covariance) matrix. An alternative to such mixture-modelling is to infer the network by thresholding the correlation matrix, e.g. at $\rho =\mathrm{1/2}$. Inferring networks like this remains popular in the most recent and high-profile studies^13^. However, thresholding correlations in this way is inevitably arbitrary and subject to user bias. The mixture-model we propose is a principled alternative that avoids those problems.

We note that our method strictly infers co-expression networks. Such networks may include transitive edges or edges which are otherwise due to indirect genomic interactions, rather than edges which specifically correspond to regulatory interactions. To build a model of a gene regulatory network, chromatin binding data and preferably also epigenomic data would need to be included. Therefore, we will include such data in the next stages of the development of our methodology. It has also been noted recently that mRNA transcription tends to be quite ‘bursty’, and therefore that network inference can be improved by including transcriptional dynamics in the model^18^. This is another promising direction we will investigate in this next stages of this work. Other promising recent work on network inference in single-cell transcriptomic data has included information-theoretic approaches^19^; this is an alternative direction we will investigate next, for inferring our initial network structure.

In this study, we have achieve excellent results using subnetwork analysis methodology which is popular amongst network statisticians, in the context of single-cell transcriptomic data. This methodology allows detection of functional subnetwork modules within these single-cell genomic networks. There is wide applicability of the methods we propose here, due to the rapid increase in popularity of data of this type, and due to the challenges of working with single-cell data, such as zero inflation and technical noise. We therefore expect that the methods proposed here will be of much use to computational biologists and bioinformaticians.

## Methods

### Data-sets and software

The main neuro-developmental data-set^11^ which was used for assessing the methods is available from NCBI (National Center for Biotechnology Information) dbGaP (database of Genotypes and Phenotypes) under accession number phs000989.v1.p1. The additional neural data-set^14^ used for validation of biological relevance is available from GEO (gene expression omnibus) under accession number GSE67835. All processing was done using the R language and associated packages; network plots were generated using the igraph package. Scripts implementing the R functions used in this analysis to infer a network adjacency matrix from a sample correlation matrix, and to infer clusters of nodes from an adjacency matrix based on the degree-corrected stochastic blockmodel, are available from https://www.ucl.ac.uk/statistics/people/thomas-bartlett.

### Correlations

All gene-gene correlations were calculated from log-expression levels. Pearson and Spearman correlations were calculated using the cor function. SCDE-adjusted correlations were calculated as weighted Pearson correlations using the corr function in the boot package. These correlation weights were calculated based on dropout-probabilities as in the original SCDE study^3^. However the difference in our study is that the correlations are now between genes, rather than between cells. The weight ${w}_{ij}^{(l)}$ for cell *l* for the correlation between genes *i* and *j* is defined as:

2 $${w}_{ij}^{(l)}=\kappa \sqrt{(1-{p}_{d}^{(l)}({x}_{i}))(1-{p}_{d}^{(l)}({x}_{j}))},$$

where ${p}_{d}^{(l)}({x}_{i})$ and ${p}_{d}^{(l)}({x}_{j})$ are dropout probabilities estimated at log-expression levels *x*_*i*_ and *x*_*j*_ in cell *l*. The dropout probability is the probability of a zero replacing a low expression level in the data due to stochastic effects in the data-collection. These dropout probabilities are estimated using the scde package (available from *github*), and the constant *κ* is set at the recommended value of $\kappa =0.95$.

Genes were included in the correlation matrix according to how robustly they are expressed. Only those genes which are expressed (i.e., are non-zero) in greater than a threshold percentage of cells were included. We set these robustness-thresholds at 5%, 25%, 50% and 75% of cells. This thresholding leads to networks of size 8027, 3314, 1190 and 387 nodes respectively for the neuron data, and 9172, 5202, 2110, and 716 nodes respectively for the oRG data.

### Clustering and subnetwork detection

Defining correlation distance as $1-|{\rho }_{ij}|$, where ${\rho }_{ij}$ is the correlation between the expression levels of genes *i* and *j*, hierarchical clustering was done with the hclust function, based on correlation distance (). PAM clustering was done with the pam function using the cluster package, also based on correlation distance. Spectral clustering into *K* clusters was done by first finding the *K*−1 top eigenvectors using the eigs function in the rARPACK package, then carrying out *K*-means clustering in this reduced eigenspace using the cclust function in the flexclust package.

Network inference (i.e., to find a binary adjacency matrix from a correlation matrix) was carried out similarly to how we have done previously^10^, and is outlined as follows. First, the correlations ${\rho }_{ij}$ between genes *i* and *j* are transformed to standard-normal variables ${z}_{ij}$, $i\in \mathrm{\{1,}\,\mathrm{...,}\,n\}$, $j\in \mathrm{\{1,\; ...,}\,n\}$, by Fisher transformation. Then, the following mixture model is fitted:

3 $${z}_{ij}\sim \mathrm{(1}-w)\cdot {\mathscr{N}}(\mathrm{0,}\,{\sigma }^{2})+w\cdot {\mathscr{N}}({\mu }_{ij},{\sigma }^{2}),$$

where *μ*_*ij*_ is inferred by the model-fitting, ${\sigma }^{2}=1$, and *w* is the mixture model weight. This model-fit is carried out with an empirical-Bayes method^20^ based on the prior:

4 $${f}_{{\rm{prior}}}({\mu }_{ij})=(1-w)\delta ({\mu }_{ij})+w\gamma ({\mu }_{ij}),$$

where $\gamma ({\mu }_{ij})$ is chosen as the Laplace prior as previously^10,20^. The Laplace parameter is set as $a=0.5$, except when the networks are inferred from (unweighted) Pearson correlations with a lower robustness-threshold (<50%). In these cases the correlations are affected more by the noise and zero-inflation described earlier, meaning that extra model flexibility is needed. Hence, in these cases *a* is also found during the model fitting. We note that, whilst *μ*_*ij*_ is inferred for each pair of genes *i* and *j*, the mixture weight *w* and Laplace parameter *a* are fixed for all $i\in \mathrm{\{1,\; ...,}\,n\}$, $j\in \mathrm{\{1,\; ...,}\,n\}$, by maximising the marginal likelihood. This is the emprical-Bayes aspect of the method; it allows each model-fit to ‘borrow strength’ from all the others. These empirical-Bayes model fits are done using the EbayesThresh package in R, with ${\hat{\mu }}_{ij}$ estimated as the posterior median. Based on this ${\hat{\mu }}_{ij}$ we then infer ${\hat{A}}_{ij}$ as:

5 $$\begin{array}{c}{\hat{A}}_{ij}=1\,{\rm{if}}\,|{\hat{\mu }}_{ij}| > 0\,{\rm{and}}\,|{\hat{\mu }}_{ji}| > 0,\\ {\hat{A}}_{ij}=0\,\,{\rm{otherwise}}\mathrm{.}\end{array}$$

Having estimated the binary adjacency matrix ${\hat{A}}_{ij}$, $i\in \mathrm{\{1,\; ...,}\,n\}$, $j\in \mathrm{\{1,\; ...,}\,n\}$, we fit the degree-corrected stochastic blockmodel (DCSBM) by regularised spectral clustering based on the graph Laplacian^9^. This is done by again using the eigs function in the rARPACK package and the cclust function in the flexclust package.

### Calculating the number of subnetworks to be detected

To make a fair comparison between the different clustering/subnetwork detection methods, for each of the methods we compare, we seek the same number of clusters/subnetworks for a network of given size. To estimate the optimal number of clusters which a data-set may be divided into, methods based on rigorous statistical theory exist: for example, the ‘gap statistic’^21^. However, such methods typically find the best number of clusters when using a specific clustering method. Instead, we would like to keep the same number of clusters/subnetworks when using each of several different clustering methods, so that they can be compared fairly. Therefore we use an alternative heuristic, described as follows.

The starting point for all the subnetwork detection methods we compare here is the correlation matrix. Hence, we use the correlation matrix as the basis on which to estimate the number of subnetworks to be found. We base this estimation on PCA (principle components analysis) of the correlation matrix. In a PCA decomposition of the correlation matrix, the number of significant principle components gives an estimate of the number of latent groups of variables which behave in a correlated way. For example, if we have *k* groups of variables, and within these groups the variables are strongly correlated, but between these groups the variables are independent, then a PCA decomposition of the correlation matrix of all these variables together would be expected to have *k* significant principle components. If the variables in such a correlation matrix correspond to network nodes, then we can expect these groupings of correlated variables to correspond to subnetworks. We therefore estimate the number of significant principle components in the correlation matrix, and then use this as an estimate of the number of subnetworks to find. We estimate the number of significant principle components using a scree-plot, by comparing the observed scree-plot with one obtained similarly after randomisation of the rows and then the columns of the correlation matrix. This gives us a heuristic method to calculate the approximate number of subnetworks or clusters to seek in the network, in a way which is independent of the different subnetwork detection methods.

We compare our method for inferring the optimum number of subnetworks/clusters with the gap-statistic method, to check that our method is working well. Using methods such as the gap-statistic to assess the optimum number of subnetworks/clusters has a high computational cost, and so for this comparison we choose a high robustness threshold (75%) to include genes in the analysis. To test the gap-statistic method, we do not use the Hclust-cor method, because we found that in this context together with the gap-statistic method it frequently leads to the best choice of number of clusters being equal to 1. We also do not use the DCSBM-adj method for this comparison, because it involves an extra processing step (i.e., adjacency matrix inference). Therefore, to test the gap-statistic method for comparison with our method, we use the PCA-cor and PAM-cor clustering methods.

Figure S3 shows two examples, in which the number of subnetworks is estimated using the scree-plot method described above, and also using the gap-statistic method (based on PCA-cor and PAM-cor). For the neuron data, the estimated number of clusters/subnetworks is similar in all cases (between 5 and 7 subnetworks are chosen as optimal); we also note here that the gap-statistic plots are extremely noisy. For the outer radial glia (oRG) data, the estimated number of subnetworks is the same for the scree-plot method and the gap-statistic method using PCA-cor (equal to 13). However for the gap-statistic method using PAM-cor, the estimated number of subnetworks is 18: this highlights the inherent variability of the optimum found from the gap-statistic between clustering methods. We note that an approximate estimate of the number of subnetworks/clusters should be sufficient here (as long as we use the same number for the same network size to assess all the methods). This is further justified because the main aim here is to cluster genes into subnetwork modules, and gene networks are thought to be hierarchical^22,23^ (i.e., display multi-scale properties). This means that different functional organisation is visible at different granularities, or scales. Therefore, there are likely to be several choices for a good number of clusters to divide genes into, when the network is viewed at different scales. We conclude that the scree-plot method works well in this context, as a heuristic to estimate an approximate number of subnetworks to divide the network into. Hence, for robustness-thresholds 5%, 25%, 50% and 75%, we divide the network into 75, 61, 16 and 5 subnetworks/clusters respectively for the neuron data, and 99, 93, 53, and 13 respectively for the oRG data.

### Comparison of methods

The comparison of the different methods is done using gene-set enrichment analysis (GSEA)^12^, based on the ‘transcription factor targets’ and ‘micro-RNA targets’ gene-sets available from the Broad Institute’s Molecular Signatures Database (MSigDB), downloaded on June 14^th^ and September 1^st^ 2016, respectively. For each method, each inferred subnetwork is tested for overlap with all of these gene-sets using Fisher’s exact test. The significance of these tests is adjusted for multiple hypothesis testing (using the Benjamini-Hochberg method^24^), and then the number of gene-sets significant at FDR (false discovery rate) $p < 0.05$ is summed. This sum quantifies the extent to which each method groups together co-regulated genes into subnetworks. The significance of the most significant overlap is also reported, as this quantifies how precisely, in the best case, the detected subnetwork reproduces a co-regulated module.

### Validation of biological relevance of detected subnetworks

This validation was based on an independent neural data-set^14^; genes were included in the network with robustness threshold 5%. The network was based on Pearson correlations, and was divided into 9 subnetworks. The overlaps of these subnetworks with canonical gene-sets for the cell-types included in the analysis (neurons, astrocytes, oligodendrocytes, OPCs microglia, endothelial cells) are shown in Fig. 4 for the DCSBM-adj method, and Figure S2 for the PAM-cor and PCA-cor methods.

## Electronic supplementary material

Supplementary Information
