# Supplementary material for: Single-cell Co-expression Subnetwork Analysis
Source: Sci Rep. 2017 Nov 8;7:15066. doi: 10.1038/s41598-017-15525-z (PMC5678118; doi:10.1038/s41598-017-15525-z)
Supplement: Supplementary file 1 — Supplementary Information [file 41598_2017_15525_MOESM1_ESM.pdf]

Single-cell Co-expression Subnetwork Analysis - Supplement

Thomas E. Bartlett, Sören Müller and Aaron Diaz

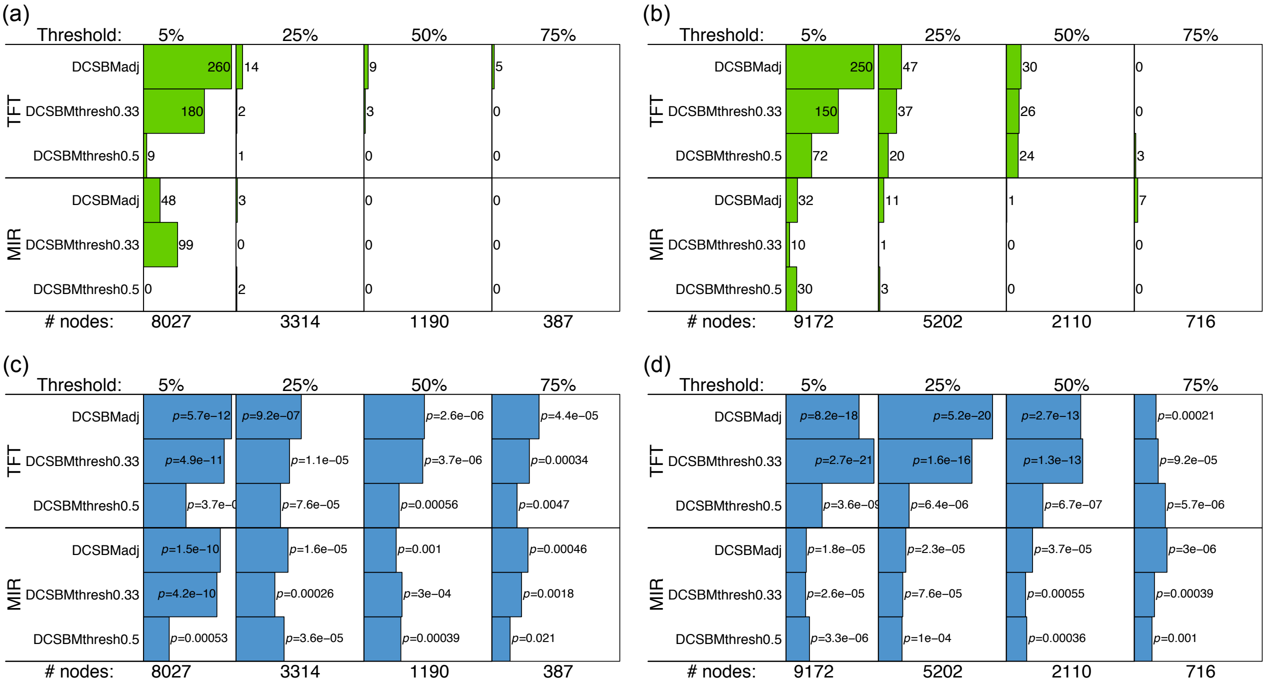

S1: **Comparison of DCSBM-adj with thresholded correlation matrices.** *The DCSBM-adj method is compared with DCSBM subnetwork inference in networks obtained from thresholded correlation matrices. This thresholding is done at* $\rho = 1/2$ *(DCSBMthresh0.5) and at* $\rho = 1/3$ *(DCSBMthresh0.33). (a) and (b) show the total number of significant overlaps (FDR* $p < 0.05$*) and (c) and (d) show the most significant overlap (*$-10\log(p)$*), between detected subnetworks and the 'transcription factor target' (TFT) and 'micro RNA target' (MIR) gene-sets, for neuron and oRG respectively. The robustness thresholds are shown above each panel (i.e., the minimum percentage of cells in which a gene must be non-zero for it to be included in the network), with corresponding numbers of network nodes below.*

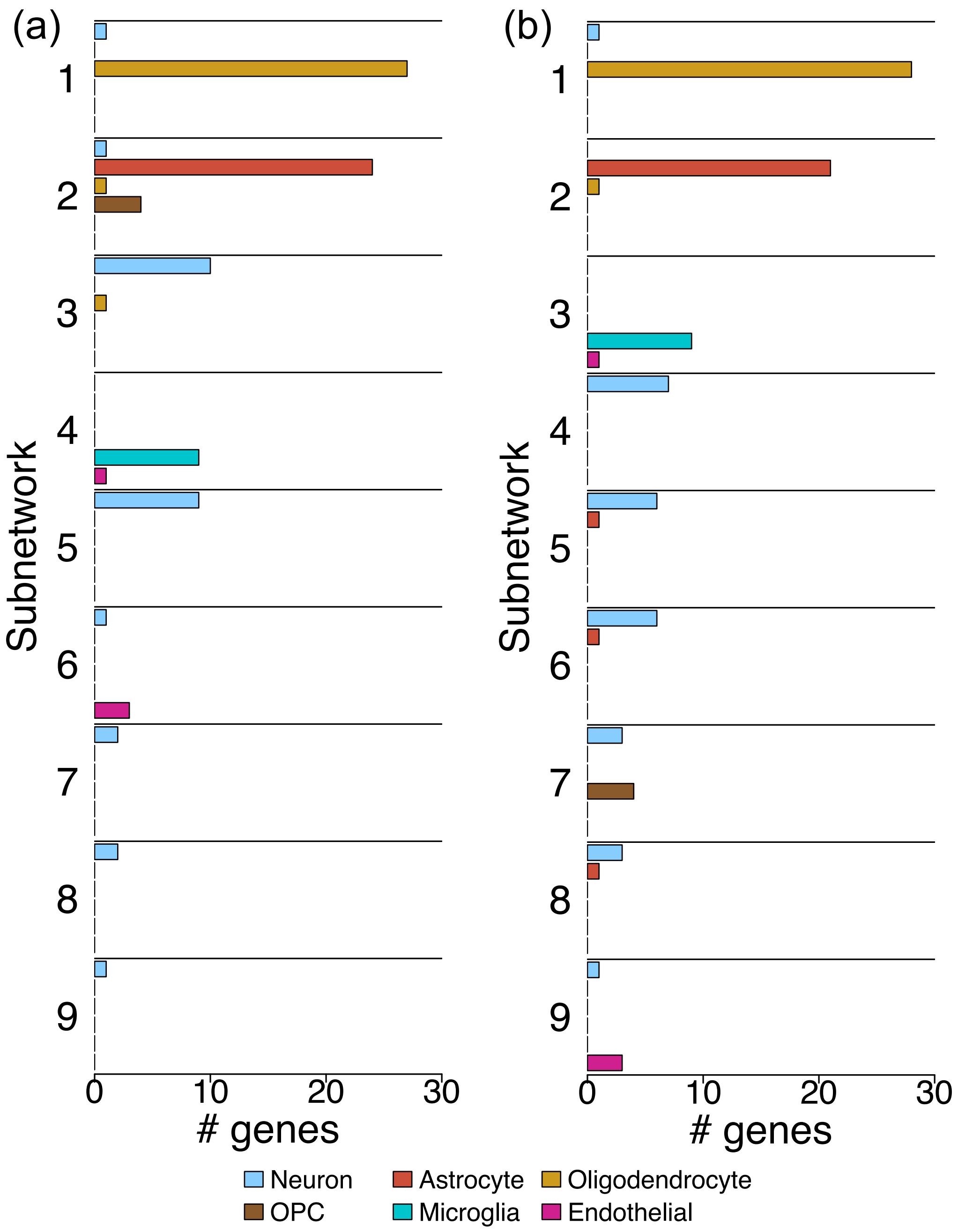

***S2:* Distribution of cell-type specific gene-sets between co-expression subnetworks.** *(a) For the PAM-cor method. (b) For the PCA-cor method.*

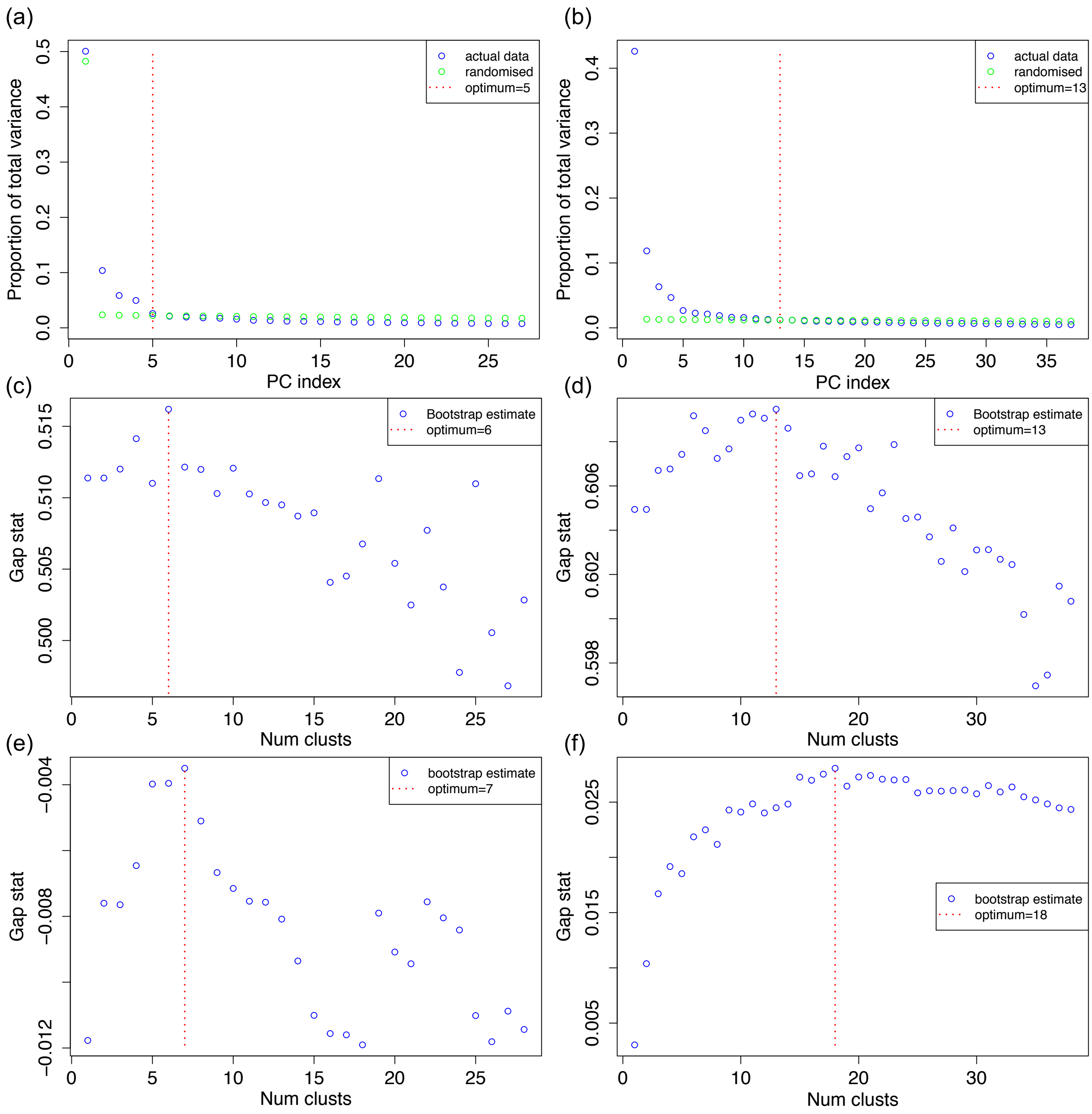

S3: **Determining number of clusters.** *(a) - (b) Scree-plots for neuron and oRG (outer radial glia) respectively. The plots show the contribution to the total variance of each principle component of the correlation matrix, together with the equivalent contributions to variance after randomising the rows and then the columns of the correlation matrix. The number of significant principle components is determined as the greatest number of components for which the observed number is greater than the equivalent obtained after randomisation. (c) - (e) The optimal number of clusters, as chosen by maximum gap-statistic: (c) neuron with PCA-cor, (d) neuron with PAM-cor, (e) oRG with PCA-cor, (f) oRG with PAM-cor.*
